# Poikilohydria, polyols, and homeoviscosity: lichen metabolomic remodeling across environmental gradients

**DOI:** 10.3389/fpls.2026.1792494

**Published:** 2026-05-04

**Authors:** Marta Pérez-Rodrigo, Patricia Moya, Clara Arenzana, Isaac Garrido-Benavent, Eva Barreno, Kristiina Mark, Ondřej Peksa, Martin Westberg, Pedro Carrasco, Francisco Marco

**Affiliations:** 1 University Institute of Biotechnology and Biomedicine (BIOTECMED), Universitat de València, Burjassot, Spain; 2 Cavanilles Institute of Biodiversity and Evolutionary Biology (ICBiBE), Universitat de València, Burjassot, Spain; 3 Department of Botany and Geology, Universitat de València, Burjassot, Spain; 4 Institute of Agricultural and Environmental Sciences, Estonian University of Life Sciences, Tartu, Estonia; 5 Museum of West Bohemia in Pilsen, Plzeň, Czechia; 6 Museum of Evolution, Uppsala University, Uppsala, Sweden

**Keywords:** arabitol, GABA, GC-MS, glycerol, lichen metabolomics, linoleic acid, mannose

## Abstract

**Introduction:**

Lichens are poikilohydric organisms whose biochemistry is strongly shaped by environmental conditions, yet their metabolome-wide adaptations across habitats remain unexplored.

**Methods:**

Here, we established a continent-scale reference for the *in-situ* metabolome of the model lichen *Ramalina farinacea*, from thalli collected across six European regions during winter and summer, using untargeted GC-MS.

**Results:**

In total, 187 small molecules were annotated, including a compact core of 12 metabolites shared across all sites and seasons. Notably, the most abundant and core metabolites have recognized osmoprotective roles. The metabolome was primarily centered on polyols: arabitol was the predominant metabolite (49.9% of total relative abundance), followed by ribitol, sucrose, sorbitol, and mannitol. Multivariate analyses revealed season, region, and climate-similarity groups as the main drivers of metabolomic dissimilarity. GABA and mannose were consistent summer markers, while linoleic acid and arabitol emerged as the top regional and climatic markers, respectively. Glycerol levels increased towards colder regimes alongside monoglycerides and mono/polyunsaturated fatty acids, reflecting patterns consistent with homeoviscous adaptation. Hierarchical clustering resolved coordinated metabolite modules that distinguish cold-continental from warm-Mediterranean regimes while preserving region-specific chemical fingerprints. Pathway over-representation analyses converged on alanine, aspartate, and glutamate metabolism. This and other differential pathways indicated coupled adjustments in carbon handling and stress physiology.

**Discussion:**

This is the first continent-scale characterization of low-molecular-weight metabolomic variation in a lichen, revealing environmentally linked metabolic remodeling beyond the traditionally studied secondary metabolites and defining new chemoenvironmental markers as the metabolic basis of poikilohydric resilience in lichens.

## 1 Introduction

Lichens are complex symbiotic associations in which a lichen-forming fungus (mycobiont) partners with one or more photosynthetic organisms (photobionts), green microalgae (phycobionts), and/or cyanobacteria (cyanobionts) to produce a metabolically integrated thallus (Honegger, 2012). Beyond these core partners, lichen thalli may host diverse communities of non-photosynthetic bacteria, protists, other fungi, and viruses, which highlight their status as miniature ecosystems (Bates et al., 2012; Hawksworth and Grube, 2020; Leiva et al., 2021; Ponsero et al., 2021). Although each partner may occur independently in nature or in axenic culture (Crittenden et al., 1995; Veselá et al., 2024), the distinctive lichen phenotype arises only through their association and reflects the symbiotic expression of the lichen-forming fungus (Honegger, 2012; Lumbsch, 2016). Due to their poikilohydric physiology (i.e., their inability to regulate internal water content), absence of roots and a protective cuticle, lichens absorb water, nutrients, and atmospheric pollutants directly across the thallus surface, strongly influencing thallus chemistry and making them sensitive, cost-effective bioindicators of air quality and climate change (Conti and Cecchetti, 2001; Thakur et al., 2024). Overall, lichens are exemplary models of symbiosis, and their definition motivates ecosystem-level questions about their metabolism and its co-adaptation (Spribille et al., 2022; Pichler et al., 2023).

The earliest isolated lichen metabolite, usnic acid, was described in 1844 during the formative years of organic and phytochemistry, effectively marking the beginning of lichen biochemistry studies (Knop, 1844; Ingólfsdóttir, 2002). Since then, several fungal-derived secondary metabolites have been characterized in lichens (Singh et al., 2024), including additional dibenzofurans (Millot et al., 2016), depsides and depsidones (Ji and Matsuda, 2025), and anthraquinones (Llewellyn et al., 2023), among others, that mediate interactions with light, water, and biota (Rundel, 1978). These compounds often vary along environmental gradients, contributing to local adaptation via photoprotection, metal tolerance, antimicrobial defense, and herbivore deterrence (Calcott et al., 2018; Schweiger et al., 2022), while exhibiting bioactivities with pharmaceutical and biotechnological potential (Ranković and Kosanić, 2015; Singh et al., 2024).

Primary metabolism in lichens, on both the photobiont and mycobiont sides, has also been investigated since the 1960s to clarify the roles of each partner in carbon exchange. Pioneering studies demonstrated the transfer of photosynthates (glucose) from the photobiont (the cyanobiont *Nostoc* Vaucher ex Bornet & Flahault) to the fungal partner in *Peltigera polydactylon* (Neck.) Hoffm., establishing a cornerstone model for primary carbon flow in lichens (Drew and Smith, 1967). Almost in parallel, research on *Xanthoria aureola* (Ach.) Erichsen identified mannitol as the principal fungal storage polyol (polyhydric alcohol with multiple hydroxyl groups), while its *Trebouxia* Puymaly phycobiont fixes carbon that accumulates and is released as the polyol ribitol (Richardson et al., 1968). Subsequent studies broadened this picture: photobiont-derived sugar alcohols (ribitol, erythritol, sorbitol) and sugars (glucose, rhamnose, sucrose) are taken up and converted by the mycobionts, which accumulate the sugar alcohols mannitol and arabitol (Spribille et al., 2022). This underscores a dynamic yet conserved carbon shuttle between partners (Pichler et al., 2023). In summary, a primary function of the photobiont is to supply the fungus with extracellular photosynthesis-derived products, mainly sugars and sugar alcohols, that sustain mutualism and the resilience of the symbiosis (Kranner et al., 2022).

*Ramalina* Ach. is a species-rich and well-studied genus of lichenized fungi (Lagreca et al., 2020; Blázquez et al., 2024). *Ramalina farinacea* (L.) Ach. forms whitish-green fruticose thalli composed of narrow and pendant sorediate laciniae. This and closely related species forming the so-called “*R. farinacea* group” (Moya et al., 2023), often develop as epiphytes on trees and shrubs, and are widely distributed throughout the northern Hemisphere, from Mediterranean to Boreal ecosystems. These mycobionts associate with the phylogenetically related microalgal species *Trebouxia jamesii* (Hildreth & Ahmadjian) Gärtner and *Trebouxia lynniae* (Barreno) (Moya et al., 2024). While thalli with *T. jamesii* occur across a broad climatic range, encompassing Mediterranean, temperate European, and Macaronesian localities, thalli with *T. lynniae* prefer warmer and more humid conditions. *R. farinacea* produces usnic, protocetraric, salazinic, and norstictic acids together with other depsidones as part of its secondary metabolism, with its composition varying between cortex and medulla, and showing a regional and altitudinal structuring of chemotypes (Arroyo and Manrique, 1988; Stocker-Wörgötter et al., 2004). In a broader chemical context within the *Ramalina* genus, other secondary metabolites have been identified, including fatty acids, sterols, and monocyclic aromatics, while carbohydrates represent the most abundant primary metabolic pool detected in chemical surveys, together with amino acids, glycolipids, glycosphingolipids, and polyols (Nascimento Moreira et al., 2015). From a physiological perspective, ribitol is known to stimulate the growth of the cultured *R. farinacea* mycobiont (Wang et al., 2009).

Here, we aimed to address a key gap in research, the limited knowledge of seasonal and biogeographic variation in lichen metabolomes, targeting the understudied low-molecular-weight compounds. Focusing primarily on *R. farinacea*, while also including a few additional specimens of closely related taxa within the “*R. farinacea* group” (*Ramalina subfarinacea* (Nyl. ex Cromb.) Nyl, *R. pollinaria* (Westr.) Ach., and a still undescribed *Ramalina* species; (Moya et al., 2023)) we tested whether metabolomic profiles differed among major biogeographic regions and climatic zones across Europe, evaluated whether there is a consistent seasonal signal across sites, and identified candidate metabolites potentially linked to environmental adaptations. By detecting and prioritizing compounds that covary with climate and habitat, we propose chemoenvironmental markers indicative of local conditions. Together, these analyses provide a continent-scale picture of lichen metabolism, linking it to geography, climate, and seasonality.

## 2 Materials and methods

### 2.1 Specimen sampling and taxonomic identification

**Figure 1:**
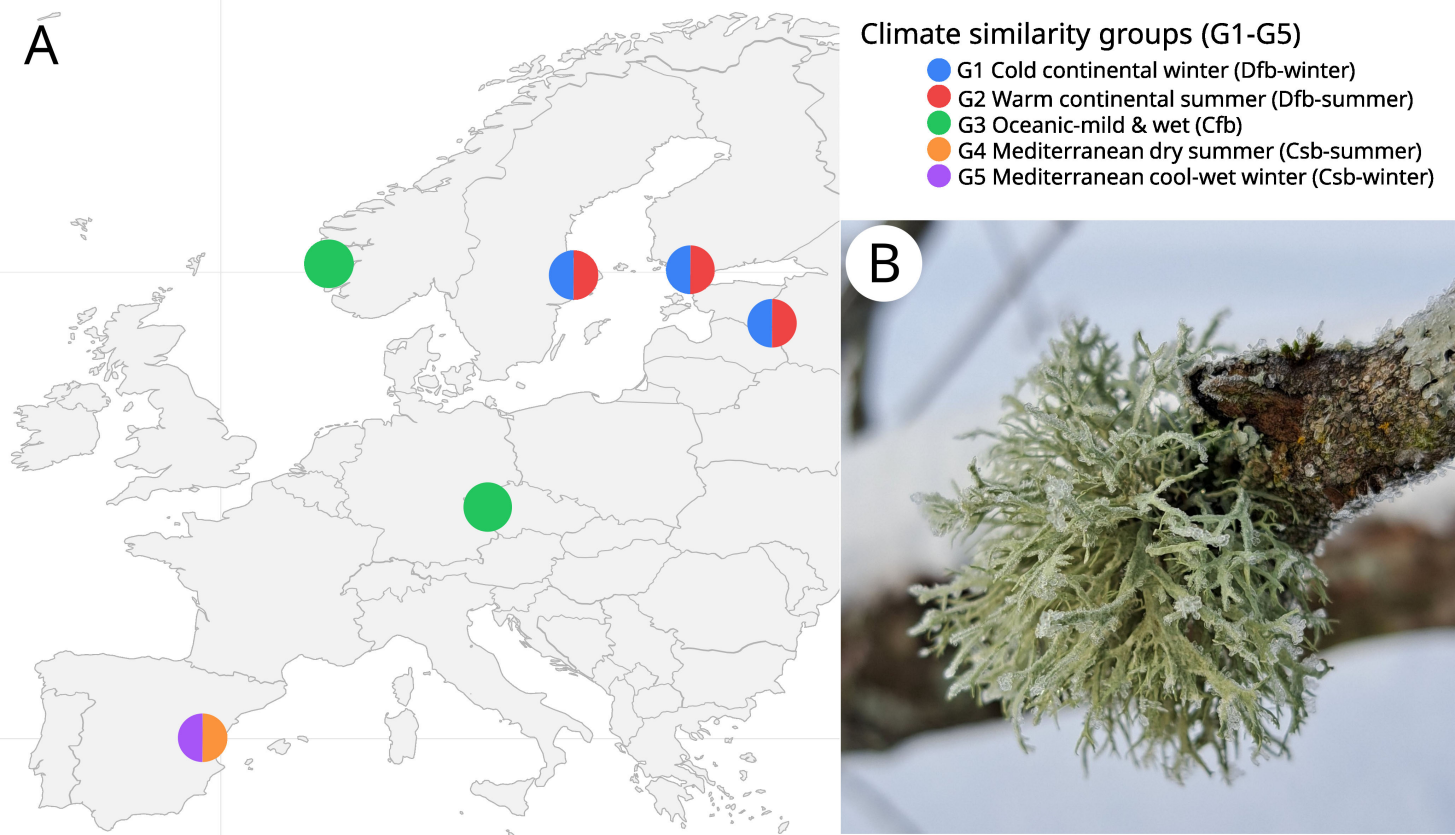
**(A)** Map of the six sampling sites of *Ramalina farinacea* specimens (n = 6 per locality and season). Point coding indicates climate similarity groups derived from the Köppen-Geiger classification (G1–G5). A more detailed interactive version of the map is provided as **Supplementary Figure S1**. **(B)** *R. farinacea* thallus collected in Tartu (Estonia; photo: Kristiina Mark).

Specimens fitting the morphological concept of *Ramalina farinacea* were collected from six sites (countries) across a geographical gradient in Europe: Czech Republic, Estonia, Finland, Norway, Spain, and Sweden (**Figure 1A**; **Supplementary Figure S1**; **Supplementary Table S1**). Sampling was carried out in winter (January-March 2023) and summer (June-July 2023), with six individuals collected per site and season. Fresh, medium-sized (≤ 5 cm in length) thalli were processed immediately after collection. Each thallus was divided into two parts: one half was air-dried and preserved at -20 °C for DNA extraction, while the other half was lyophilized overnight and stored in the dark with silica gel for metabolite analysis. For the taxonomic identification of mycobionts in the collected specimens, we considered the “*R. farinacea* group” species delimitation (Moya et al., 2023) based on phylogenetic analyses of the nuclear ribosomal Internal Transcribed Spacer region. The same marker was used to provide a molecular-based taxonomic ID for the photobiont (Moya et al., 2024). For the 30 additional thalli not included in (Moya et al., 2024), the same sampling and processing methodology described in that work was followed. The corresponding identifications are shown in **Supplementary Table S1**.

### 2.2 Sample preparation and metabolome determination by untargeted GC-MS

Metabolites were extracted and derivatized according to a protocol for plant samples (Lisec et al., 2006), with major modifications. First, thallus fragments were randomly pooled per lichen specimen to obtain 15 mg dry weight (DW), weighed on a precision balance. Then, lichen material was finely ground with approximately 300 mg glass beads (500-750 μm diameter) using a vortex with a bead-beating attachment (Vortex-Genie 2) at maximum speed for 15 min. A volume of 1.4 ml of HPLC-grade methanol (pre-cooled to -20 °C) and 350 μl of antipyrine stock solution (10 mM in methanol, used as the internal standard, IS) were added to the ground material. Samples were incubated at 70 °C with shaking (950 rpm, 10 min), centrifuged (11,000 g, 10 min), and 1.2 ml of the supernatant was transferred to PCR clean purity grade tubes. A 400 μl aliquot of the extract was then dried in a SpeedVac for chemical derivatization, while the remainder volume was stored at -20 °C for future use.

Derivatization involved methoxyamination followed by silylation of the dried samples, with an empty tube processed in parallel as a blank control. For methoxyamination, 40 μl of freshly prepared methoxyamine hydrochloride (20 mg/ml in pyridine) were added, and samples were incubated at 37 °C (650 rpm, 2 h). For silylation, 70 μl of *N*-methyl-*N*-(trimethylsilyl)trifluoroacetamide (MSTFA) were added, along with 5 μl of C7-C30 saturated alkanes (1 mg/ml) to the first and last samples of each batch for retention index calibration (van Den Dool and Kratz, 1962). Samples were incubated at 37 °C (650 rpm, 30 min) and transferred to glass-insert vials for Gas Chromatography-Mass Spectrometry (GC-MS) analyses.

GC-MS analyses were performed on an Agilent 5977A mass spectrometer with a quadrupole analyzer and an HP-5MS UI capillary column (30 m × 0.25 mm × 0.25 μm; Agilent 19091S-433UI). Helium was used as the carrier gas (1 ml/min, constant flow). The injector was operated in splitless mode (79.6 ml/min, 1 min), and the injection volume was 2 μl. The temperature program was as follows: 60 °C (5 min), ramped at 10 °C/min to 250 °C (5 min), then 20 °C/min to 300 °C (10 min). The transfer line, ion source, and quadrupole temperatures were set to 280 °C, 230 °C, and 150 °C, respectively. Mass spectra were acquired in electron impact mode (70 eV), with a scan range of 30–650 m/z and a solvent delay of 8 min. Each sample was injected in duplicate as technical replicates.

Peak deconvolution, spectral matching, and integration were performed using MassHunter GC/MS Acquisition and MassHunter Qualitative software (Agilent Technologies). C7-C30 alkanes were used for peak alignment. Metabolites were annotated by comparing spectra with the NIST commercial library (Ausloos et al., 1999) and manually validated against the Golm Metabolome Database (GMD; (Kopka, 2006)). Abundance values were normalized to the IS by applying a scaling factor to each sample, calculated as fj=median(IS)/ISj and multiplying it by all peak areas in that sample. Pure standards of selected metabolites were analyzed for method validation, confirming annotations and supporting relative abundance assessment.

### 2.3 Data analysis

Sampling site maps were created in Python v. 3.13 using the *Folium* library (v. 0.20) with the *Leaflet* JavaScript library (v. 2.0) for the interactive version, and in R v. 4.5 (R Core Team, 2025) using the packages *sf* (v. 1.0-21; (Pebesma et al., 2025)), *rnaturalearth* (v. 1.1.0; (Massicote et al., 2025)), and *rnaturalearthdata* (v. 1.0; (South et al., 2025)) for the static version. Sampling sites were classified according to Köppen-Geiger climatic zones (Beck et al., 2018) and further grouped into climate similarity groups (G1-G5) based on location-specific long-term-averaged climate data. Additional bioregional classifications followed (Rivas-Martínez et al., 2017; Moya et al., 2024; European Environment Agency (EEA), 2025).

Metabolite data were processed and analyzed using R software v. 4.5 (R Core Team, 2025). Permutational multivariate analysis of variance (PERMANOVA), distance-based redundancy analysis (dbRDA) and variance partitioning were performed using the packages *vegan* (v. 2.7-1; (Oksanen et al., 2025)) and *adespatial* (v. 0.3-28; (Dray et al., 2012)) to test the significance of ecological and geographical factors in explaining overall metabolome variation and quantify unique/shared variance. PERMANOVA and dbRDA models included the geographic and ecological classifications described above, together with region, season, phorophyte species, latitude, altitude, and distance to the sea as explanatory factors. For variance partitioning analysis, explanatory variables were grouped into four categories: environment (altitude, distance to sea), climate (season, Köppen-Geiger classification, climate similarity groups), space (spatial structure, region), and host (phorophyte).

Principal component analysis (PCA) was conducted using *factoextra* (v.1.0.7; (Kassambara and Mundt, 2020)) as an exploratory unsupervised method to detect overall patterns in the normalized dataset. Partial Least Squares-Discriminant Analysis (PLS-DA) was carried out with *mixOmics* (v. 6.32; (Rohart et al., 2017)) as a supervised approach to maximize separation between predefined significant groups identified by PERMANOVA and dbRDA. Variable Importance in Projection (VIP) scores were calculated, and only compounds with a False Discovery Rate (FDR) < 0.05 and VIP ≥ 1.0 were considered significantly different between groups. Model performance was evaluated using receiver operating characteristic (ROC) curves and area under the curve (AUC) metrics, calculated with *pROC* (v.1.19; (Robin et al., 2025)). Heatmaps of confusion matrices (5-fold cross-validation, row-normalized) were visualized with *pheatmap* (v. 1.0.13; (Kolde, 2025)).

Intersections and shared metabolite composition were visualized with UpSet diagrams generated using the Intervene web server (Khan and Mathelier, 2017). Additional heatmaps for the significantly different metabolites were constructed using the Morpheus web server (Morpheus, n.d.). Finally, a metabolic pathway over-representation analysis was performed on the significantly different metabolites annotated in the KEGG pathway database (Kanehisa et al., 2025) using the *KEGGREST* package (v. 1.48.1; (Tenenbaum, 2025)). The background set comprised all detected compounds with KEGG identifiers. Enrichment was tested with one-sided Fisher’s exact tests, and p-values were adjusted for multiple testing using the Benjamini-Hochberg method to obtain q-values. Analyses were restricted to plant and fungal annotated pathways.

## 3 Results

### 3.1 Species identity of symbiotic partners in *R. farinacea*

Mycobiont identification confirmed *R. farinacea* s. str. in most samples (66 out of 72 individuals). One specimen of *R. subfarinacea*, four *Ramalina* sp. (*sensu* (Moya et al., 2023)) and one *R. pollinaria* were also included in the dataset. The dominant photobiont identified in all analyzed thalli (**Figure 1**; **Supplementary Figure S1**; **Supplementary Table S1**) was *Trebouxia jamesii*.

### 3.2 *In situ* metabolome of *R. farinacea*: arabitol dominance, and other core and bioactive compounds

**Figure 2:**
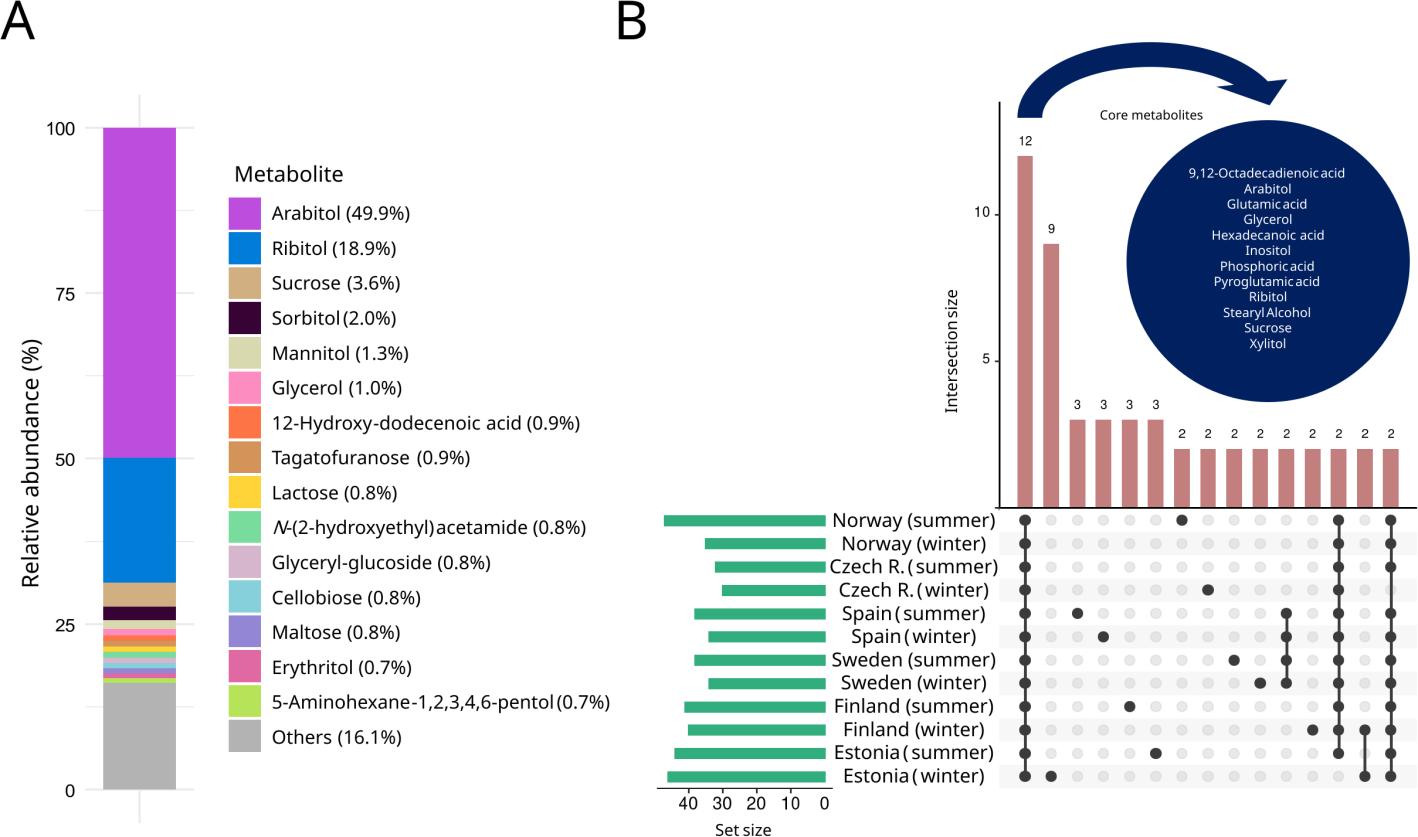
**(A)** Average metabolomic profile of *R. farinacea*. The 15 most abundant compounds are shown in terms of their relative proportion (%) of the total metabolome. **(B)** UpSet diagram illustrating the distribution and overlap of detected metabolites among the analyzed groups. The upper bar plot illustrates the number of shared metabolites, with connected dots indicating the corresponding sets. Bars on the left show the total number of metabolites per group. The blue circle highlights the set of metabolites consistently present in all samples (core metabolome).

The seasonally and geographically stratified sampling enabled the characterization of the representative *in situ* metabolome of *Ramalina farinacea* throughout Europe (**Figure 2A**). Untargeted GC-MS profiling revealed the 187 small-molecule annotated metabolites. Among them, arabitol dominated, accounting for 49.9% of total relative abundance, followed by ribitol (18.9%), both far exceeding all other compounds. The next most abundant were sucrose (3.6%), sorbitol (2.0%), and mannitol (1.3%). The 15 major compounds comprised six polyalcohols (arabitol, ribitol, sorbitol, mannitol, glycerol, and erythritol), five sugars (sucrose, tagatofuranose, lactose, cellobiose and maltose), the fatty acid 12-hydroxy-dodecenoic acid, the glycosidic conjugate glyceryl-glucoside, the amino alcohol derivative *N*-(2-hydroxyethyl)acetamide, and the amino sugar alcohol 5-aminohexane-1,2,3,4,6-pentol.

Under the season-by-region grouping definition (**Figure 2B**), *R. farinacea* sampled in Norway during summer showed the widest metabolite diversity (47 annotated metabolites), whereas samples from the Czech Republic in winter showed the lowest (30). The core metabolome, defined as metabolites consistently detected across all sites and both seasons, comprised twelve compounds (**Figure 2B**): arabitol, ribitol, xylitol (the three pentitol stereoisomers), glycerol, inositol, sucrose, 9,12-octadecadienoic acid (linoleic acid), hexadecanoic acid, stearyl alcohol, glutamic acid, pyroglutamic acid (5-oxoproline), and phosphoric acid.

Beyond these, several other metabolites of physiological or biotechnological interest were detected (**Supplementary Table S2**). Additional sugar alcohols included threitol, dulcitol, and galactinol. A wide range of fatty acids was also annotated, including eicosapentaenoic (omega-3 PUFA), stearic (SFA), and oleic (MUFA) acids, together with derivatives such as 3-hydroxydodecanedioic and 2-hydroxyoctanoic acids. Other noteworthy compounds included sulfur-containing metabolites (2-thiophenethiol and 2,4-di-*tert*-butylphenol), the aliphatic alcohol cuminyl alcohol, γ-tocopherol (vitamin E), and 1-monopalmitin (a monoacylglyceride/glycosylglycerol). Finally, diverse sugars, amino acids, and other organic acids involved in central metabolism and specialized metabolism were also annotated.

### 3.3 Season, region, and climatic group as metabolome shaping factors

The broad sampling of *Ramalina farinacea* enabled the analysis of the factors that significantly structure metabolomic dissimilarities across the studied environmental range (**Supplementary Table S1**). PERMANOVA (**Supplementary Figure S2A**), variance partitioning analysis (**Supplementary Figure S2B**), and dbRDA (**Table 1**) analyses consistently identified season, region, and climate similarity group (Köppen-Geiger derived, G1-G5) as the primary drivers of metabolomic variation.

**Table 1:** Summary of model selection results from distance-based redundancy analysis (dbRDA) assessing ecological and geographical factors structuring metabolomic variation in *Ramalina farinacea*.

dbRDA	Df	Variance	F	Pr (>F)	Signif.
Season	1	5.616	5.408	0.001	***
Region	5	59.889	11.534	0.001	***
Climate similarity group (G1-5)	2	7.932	3.819	0.001	***
Phorophyte	2	2.344	1.129	0.253	–
Residual	129	133.961	–	–	–

Tested factors included region, season, Köppen-Geiger climatic zone, climate similarity groups derived from the Köppen-Geiger classification (G1–G5), bioregions (Rivas-Martínez et al., 2017; Moya et al., 2024; European Environment Agency (EEA), 2025), phorophyte, latitude, altitude, and distance to the sea. Df, degrees of freedom; F, F statistic; Pr(>F), permutational p-value.

According to PERMANOVA (**Supplementary Figure S2A**), these three factors had significant marginal effects, with the climate similarity group accounting for the largest proportion of variance in the distance matrix (R² = 0.037). The remaining factors, including biogeographic regions (Rivas-Martínez et al., 2017; Moya et al., 2024; European Environment Agency (EEA), 2025), Köppen-Geiger climatic zones, phorophyte, latitude, altitude, and distance to sea, did not significantly explain metabolomic differences. However, several of these variables exhibited partial collinearity with the three leading factors.

Variance partitioning (**Supplementary Figure S2B**) attributed more than one-third (35%) of the total variance to the modeled components, while 65% remained unexplained. Among independent fractions, climate (5%) and space (4%) accounted for the largest contribution, whereas a negligible contribution (0%) was attributed to phorophyte and continuous environmental variables (altitude, distance to sea). Shared fractions were non-trivial, including an 8% joint component reflecting covariance among space, climate, and host.

dbRDA analysis (**Table 1**) confirmed the full model as highly significant (F = 7.30, p = 0.001), explaining ca. 35% of total variance. Among the evaluated factors, geographic region contributed the largest fraction of variance (F = 11.53, p = 0.001), followed by season (F = 5.41, p = 0.001) and climate similarity group (F = 3.82, p = 0.001). In contrast, phorophyte showed no significant effect (p = 0.253). Additional predictors were excluded during model selection due to collinearity, indicating redundancy among alternative environmental classifications.

### 3.4 Sample diversity and discriminant metabolites

**Figure 3:**
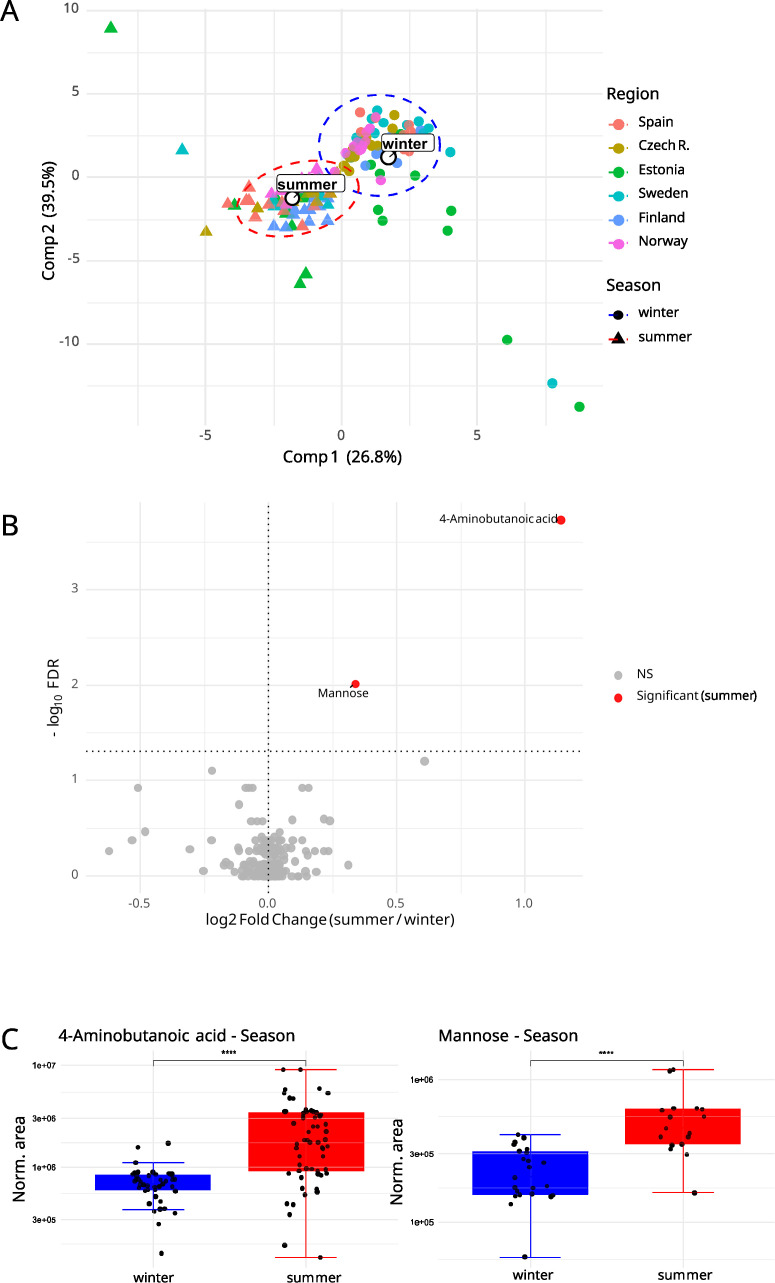
**(A)** Global discrimination of *ramalina farinacea* metabolomes using partial least squares discriminant analysis (PLS-DA) by season. Ellipses indicate the 95% confidence intervals for each group, and central points denote group centroids. **(B)** Metabolites contributing significantly to season differentiation (VIP score mean ≥ 1 for components 1 and 2, and FDR ≤ 0.05) are displayed in a volcano plot (n = 2 metabolites). **(C)** Boxplot showing the distribution of normalized peak areas for the two significant metabolites in the season group model, 4-aminobutanoic acid and mannose.

**Figure 4:**
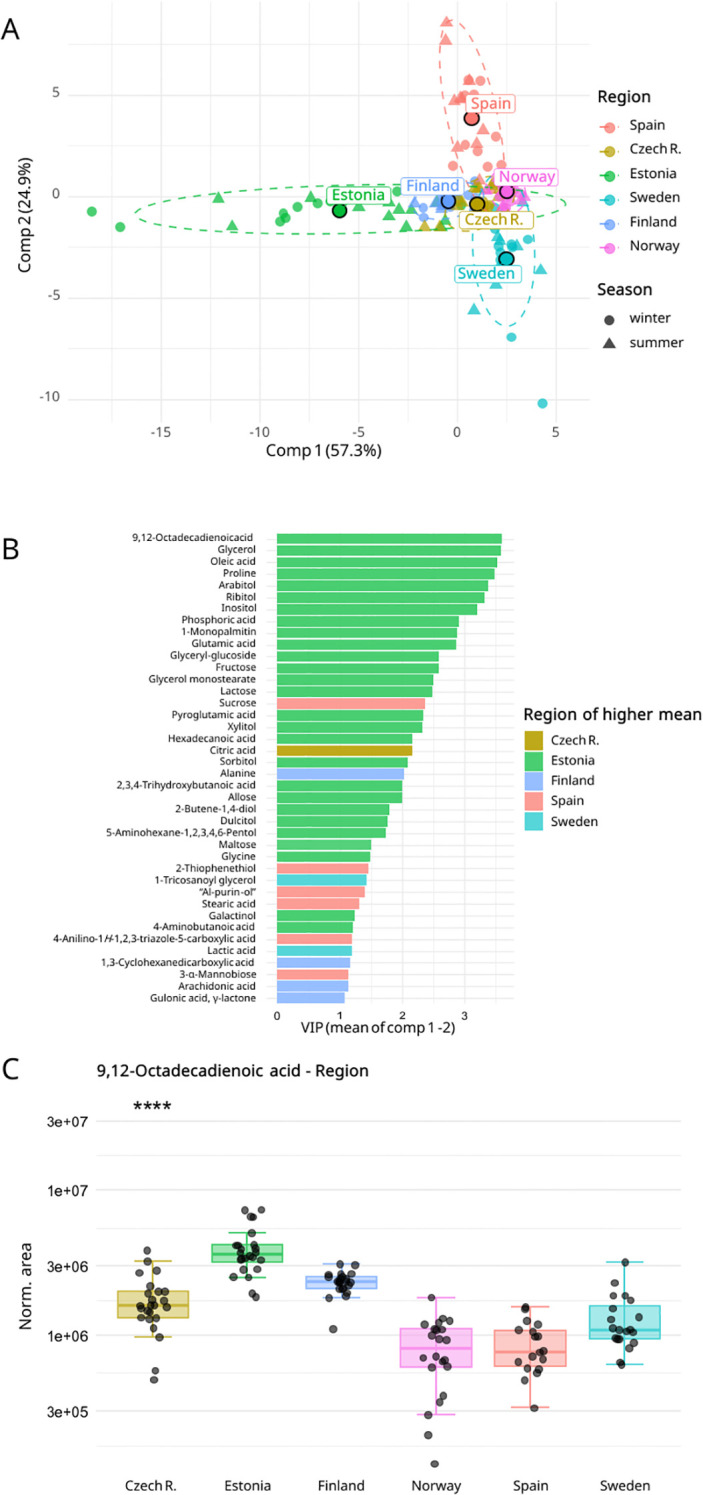
**(A)** global discrimination of *ramalina farinacea* metabolomes using partial least squares discriminant analysis (PLS-DA) by region. Ellipses indicate the 95% confidence intervals for each group, and central points denote group centroids. **(B)** Metabolites contributing significantly to region differentiation (VIP score mean ≥ 1 for components 1 and 2, and FDR ≤ 0.05) are displayed in a barplot (n = 40 metabolites). The abbreviation “AI-purin-ol” refers to *4-amino-8-(hydroxymethyl)-6a,7,8,9a-tetrahydro-5H-furo[3′,2′:4,5]imidazo[1,2-e]purin-7-ol*. **(C)** Boxplot showing the distribution of normalized peak areas for the most significant metabolite in the region group model, 9,12-octadecadienoic acid.

**Figure 5:**
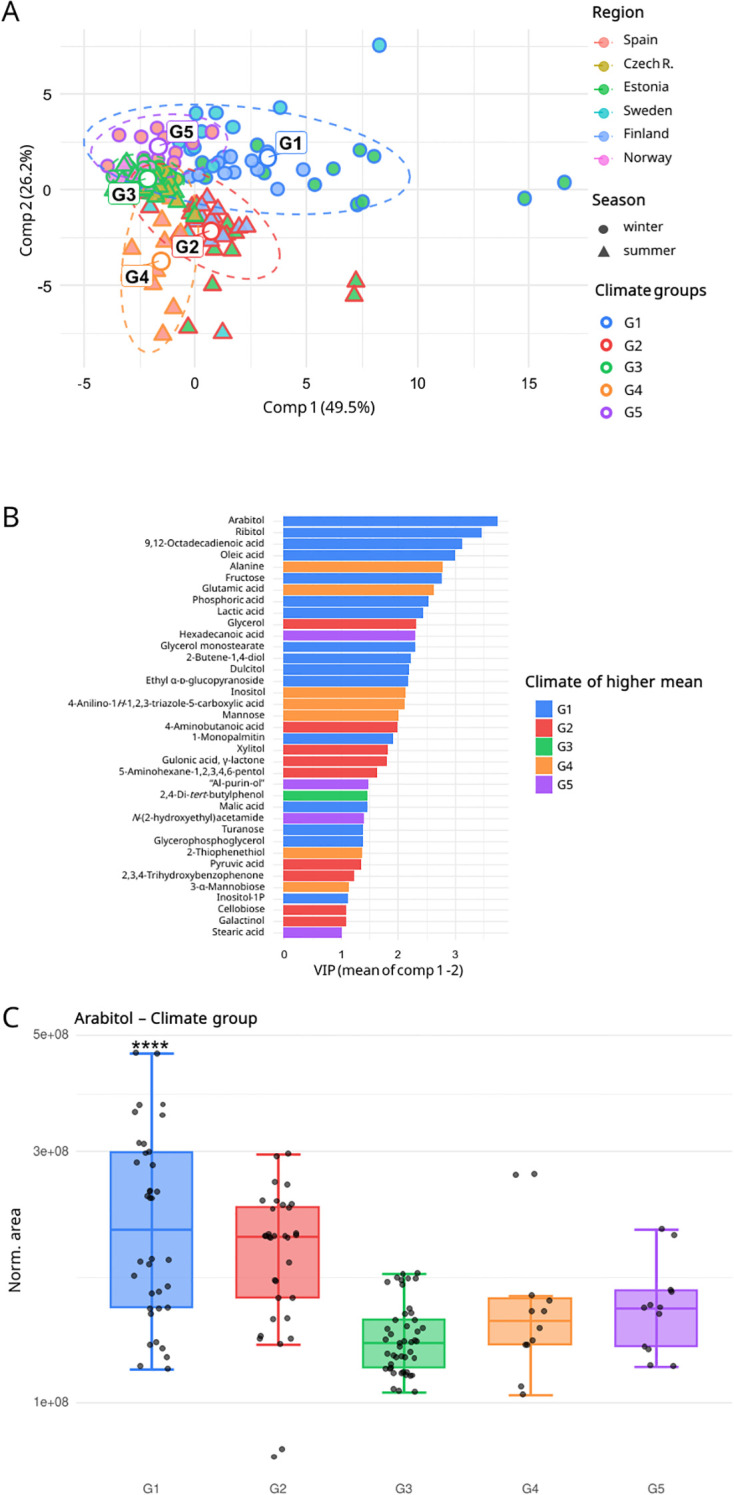
**(A)** Global discrimination of *Ramalina farinacea* metabolomes using Partial Least Squares Discriminant Analysis (PLS-DA) by climate group. Ellipses indicate the 95% confidence intervals for each group, and central points denote group centroids. **(B)** Metabolites contributing significantly to climate differentiation (VIP score mean ≥ 1 for components 1 and 2, and FDR ≤ 0.05) are displayed in a barplot (n = 37 metabolites). The abbreviation “AI-purin-ol” refers to *4-amino-8-(hydroxymethyl)-6a,7,8,9a-tetrahydro-5H-furo[3′,2′:4,5]imidazo[1,2-e]purin-7-ol*. **(C)** Boxplot showing the distribution of normalized peak areas for the most significant metabolite in the climate group model, arabitol.

Unsupervised multivariate analysis by PCA of samples by region and season (**Supplementary Figure S3**) revealed grouping of *Ramalina farinacea* with considerable overlapping. To further resolve this metabolomic differentiation, supervised multivariate analyses by PLS-DA were performed considering the three significant differentiating environmental factors: season (**Figure 3A**), region (**Figure 4A**), and climate group (**Figure 5A**).

The PLS-DA season model showed a clear separation of metabolomic profiles between summer and winter samples (**Figure 3A**), independent of the sampling site. Separation along the two principal components explained 26.8% and 39.5% of the variance, respectively, and was evident within each region (**Supplementary Figure S4A**). ROC curves with corresponding AUCs (**Supplementary Figure S4B**) indicated excellent discrimination of the season model, with optimal specificity and sensitivity and AUCs close to 1. Confusion matrix heatmaps from five-fold cross-validation (**Supplementary Figure S4C**) further supported the model classification power. To identify season markers, metabolites were ranked by the mean VIP across the first two latent components, with significance assessed by FDR-controlled tests. Applying the *a priori* threshold (mean VIP ≥ 1 and FDR ≤ 0.05), only two metabolites, 4-aminobutanoic acid (GABA) and mannose, met both criteria. Each showed higher relative abundance in summer in the pooled analysis (**Figures 3B, C**).

The regional model (**Figure 4A**) revealed a clear separation along the two principal components of the PLS-DA (57.3% and 24.9% variance). This pattern persisted when examined within each season (**Supplementary Figure S4D**). Discriminative performance was strongest for specimens collected in Sweden, Spain, Estonia, and Norway (high ROC-AUC, well-calibrated matrices; **Supplementary Figures 4E, F**), whereas *R. farinacea* from Czech Republic and Finland showed moderate separability (AUC ≈ 0.65 and low diagonal accuracy in the confusion matrix). Furthermore, forty region marker metabolites met the predefined significance criteria (**Figure 4B**). Estonia exhibited the highest relative abundances for most discriminants and the greatest dispersion along component 1 (**Figure 4A**). The top regional marker was 9,12-octadecadienoic acid (**Figure 4C**).

Finally, the climate-group model (**Figure 5A**) revealed a separation along the two principal components of the PLS-DA of 49.5% and 26.2%. This pattern remained consistent across regions (**Supplementary Figure S5A**) and seasons (**Supplementary Figure S5B**). Discriminative performance exceeded that of the regional model, with high specificity and sensitivity and AUC > 0.9 for all groups except G2 and G3 (AUC = 0.882 and 0.896); confusion matrices were well-calibrated, except for G3 and G5 (**Supplementary Figures 5C, D**). Moreover, thirty-seven metabolites met the predefined criteria as climate-group markers (**Figure 5B**). This model yielded a slightly smaller set of significant compounds than the regional analysis, with substantial overlap between the two. The G1 group (“cold continental winter” group) exhibited the highest relative abundances for the higher number of discriminant metabolites. Among all metabolites, arabitol emerged as the top climate-group marker (**Figure 5C**).

### 3.5 Functional reorganization of metabolism across regions and climate groups

**Figure 6:**
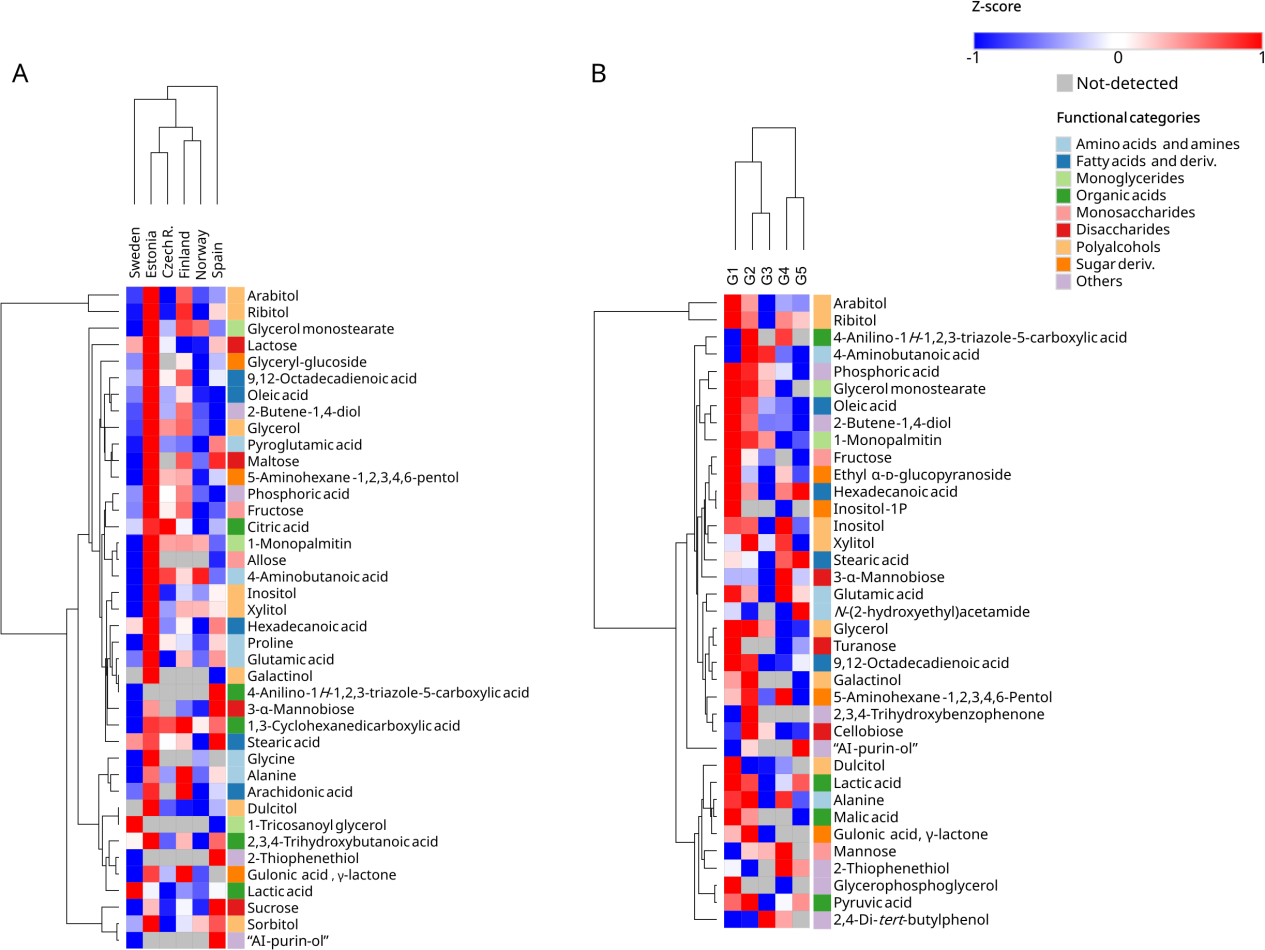
Hierarchical clustering heatmaps of significant metabolites across **(A)** regions and **(B)** climate groups, respectively, tagged by functional categories. Columns were clustered using one minus Pearson correlation, and rows using Euclidean distance, both with average linkage. Data were log_2_-transformed and standardized as z-scores per metabolite. The abbreviation “AI-purin-ol” refers to *4-amino-8-(hydroxymethyl)-6a,7,8,9a-tetrahydro-5H-furo[3′,2′:4,5]imidazo[1,2-e]purin-7-ol*. Metabolites were considered significant when the mean VIP score for components 1 and 2 was ≥ 1 and FDR ≤ 0.05.

Analysis of metabolites differing across regions and climatic groups revealed patterns of metabolic adaptation to environmental conditions. At the regional level (**Figure 6A**), clustering based on differential metabolite abundance resolved four groups: (1) specimens of *Ramalina farinacea* from Spain showed the most distinct composition, (2) followed by Swedish specimens, whereas (3) Estonian and Czech, and (4) Finnish and Norwegian specimens clustered together. Clustering by climate similarity group (**Figure 6B**) yielded an even clearer structure: G1-G2-G3 (northern/colder regimes, i.e., continental winter and summer, and oceanic-mild and wet) grouped together, whereas G4-G5 (southern/warmer regimes, i.e., Mediterranean dry summer and cool-wet winter) formed a second cluster.

Hierarchical clustering also revealed modules of co-varying metabolites. The two main clusters consistently differentiate arabitol and ribitol from the rest of the metabolites in both models (**Figure 6**). Under the regional classification (**Figures 4B**, **6A**), several metabolites showed region-biased enrichment. Many overlapped with the climate-group discriminants, but region-specific differentiators were also evident. Notably, Estonian specimens displayed the highest relative abundance of most metabolites, being specially enriched in dulcitol, inositol, galactinol, glyceryl-glucoside, oleic acid, allose and glycine; Swedish *R. farinacea* in 1-tricosanoyl glycerol and lactic acid; Spanish samples in 4-anilino-1*H*-1,2,3-triazole-5-carboxylic acid, 4-amino-8-(hydroxymethyl)-6a,7,8,9a-tetrahydro-5*H*-furo[3′,2′:4,5]imidazo[1,2-*e*]purin-7-ol, sucrose and 2-thiophenethiol; and Finnish specimens in alanine. In *R. farinacea* thalli collected in the less differentiated regions (Norway and the Czech Republic) (**Figure 4A**), differential metabolites were comparatively less abundant, with only citric acid showing a regional maximum in Czech R. arabitol, the dominant metabolite (**Figure 2A**), showed significant regional differences, peaking in Estonian and Finnish samples, similarly to its isomer ribitol.

Under the climate classification (**Figures 5B**, **6B**), several compounds displayed a cold-to-warm gradient, with higher abundance in colder regimes and decreasing toward warmer ones. These included the abundant arabitol, monoglycerides (1-monopalmitin and glycerol monostearate), oleic acid, glycerol, 2-butene-1,4-diol, and phosphoric acid (phosphate). Some metabolites showed preferential accumulation in the continental regimes: fructose, 9,12-octadecadienoic acid, galactinol, malic acid, and gulonic acid δ-lactone accumulated strongly in G1-G2. Within the coldest group (G1), we observed significant enrichment of dulcitol, turanose, and the phosphate derivatives glycerophosphoglycerol and inositol-1-phosphate. By contrast, G2 (continental summer) was characterized by elevated 2,3,4-trihydroxybenzophenone and cellobiose. Other compounds peaked at both climatic extremes (continental G1-G2 and Mediterranean G4-G5) but were reduced in the milder G3. These include ribitol, stearic acid, hexadecenoic acid, glutamic acid, pyruvic acid, lactic acid, and alanine. Ethyl α-D-glucopyranoside peaked in both extremes (G1 and G4), while xylitol peaked in the two summers. In the Mediterranean regimes (G4-G5), 2-thiopentitol was also prominent. By contrast, 3-α-mannobiose peaked in the Mediterranean summer (G4), while *N*-(2-hydroxyethyl)acetamide was abundant in the Mediterranean winter (G5). 4-anilino-1*H*-1,2,3-triazole-5-carboxylic acid and 5-aminohexane-1,2,3,4,6-pentol were summer-enriched in both G2 and G4. By contrast, the previously identified summer markers GABA and mannose reached higher levels in G2 and G3, with mannose also elevated in G4. Additional features include inositol, elevated in G4 followed by continental regimes, 2,4-di-*tert*-butylphenol, which was most abundant in G3 and G4, and 4-amino-8-(hydroxymethyl)-6a,7,8,9a-tetrahydro-5*H*-furo[3′,2′:4,5]imidazo[1,2-*e*]purin-7-ol, which peaks in the Mediterranean winter (G5) with a secondary maximum in G2.

### 3.6 Pathway-level reorganization

**Figure 7:**
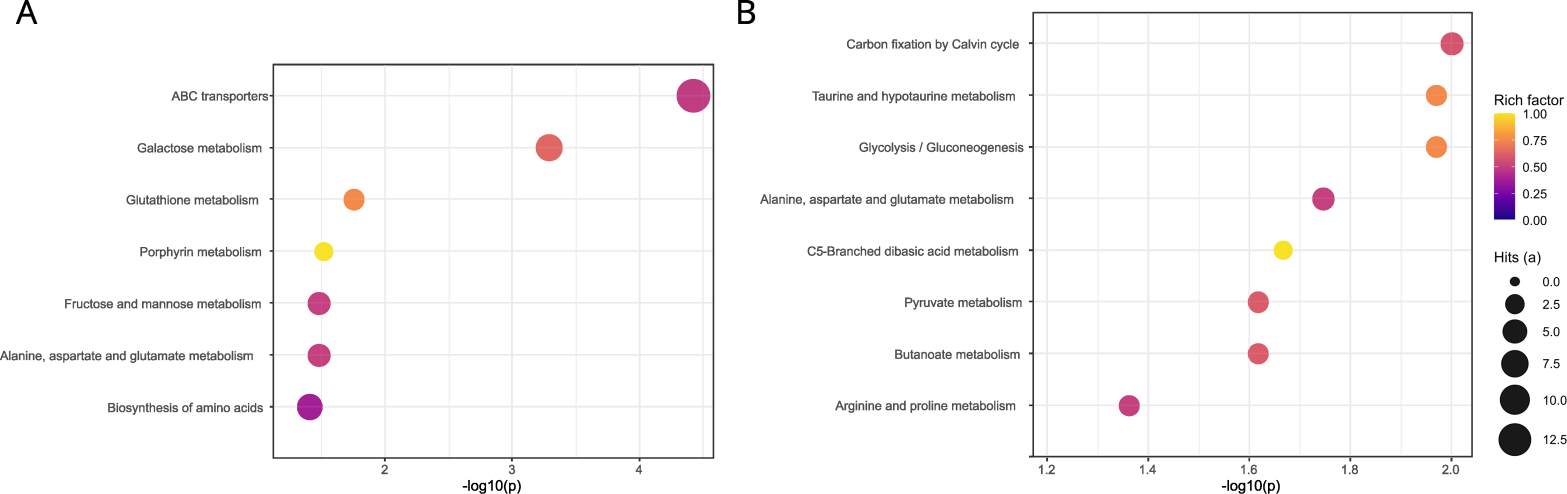
Significantly enriched KEGG pathways by p-value by **(A)** region and **(B)** climate group. The x-axis shows -log10(p) (one-sided Fisher’s exact tests; considered significant when p-value < 0.05); point color encodes the rich factor (a/(a+c)) and point size encodes hits (a). The background is all detected compounds with KEGG IDs. Only plant and fungal pathways were included.

Pathway enrichment analysis on the differential compounds revealed metabolic pathways associated with specific regions and climatic groups (**Figure 7**). Across both classification analyses, alanine, aspartate, and glutamate metabolism was the only shared, significantly enriched pathway. Differences between models also emerged: for region (**Figure 7A**), the enriched pathways additionally included ABC transporters, galactose, glutathione, porphyrin, fructose and mannose metabolisms, and biosynthesis of amino acids; whereas for climate groups (**Figure 7B**), carbon fixation by Calvin cycle, taurine and hypotaurine metabolism, glycolysis/gluconeogenesis, C5-branched dibasic acid metabolism, pyruvate metabolism, butanoate metabolism, arginine and proline metabolism were differential.

## 4 Discussion

The continent-scale survey of the lichen *Ramalina farinacea* reveals a metabolome that is conservative in its core composition yet environmentally responsive to seasonal, regional, and climatic variation. The metabolome is dominated by the five-carbon polyol arabitol, which accounts for half of the total relative abundance (**Figure 2A**). This is consistent with the carbon transfer model proposed in other lichen symbioses (Spribille et al., 2022), where the photobiont, here *T. jamesii*, delivers a photosynthesis-derived sugar alcohol, probably ribitol (our second-most abundant), to *R. farinacea* mycobiont, which converts and stores it as arabitol.

The predominance of arabitol has also been reported in other *Ramalina* species, including *R. yasudae* Räsänen, *R. fraxinea* (L.) Ach., *R. menziesii* Taylor, *R. calicaris* (L.) Röhl., *R. sinensis* Jatta and *R. siliquosa* (Huds.) A.L. Sm (Kosugi et al., 2013; Nascimento Moreira et al., 2015). With arabitol dominating, followed by ribitol, and the far less abundant sorbitol, mannitol, and glycerol (**Figure 2A**), *R. farinacea* exhibits a polyol-based carbon economy that points to robust interpartner carbon transfer across the analyzed range of field conditions.

Season, region, and climate similarity groups were identified as the leading factors structuring metabolomic dissimilarities (**Table 1**; **Supplementary Figure S2**). Climate groups summarized broad environmental regimes that were both season and region-dependent, while regions retained distinctive chemical fingerprints consistent with local context. Previous studies have examined how environmental gradients influence lichen secondary metabolites (dibenzofurans, depsides, depsidones, anthraquinones, etc.) (Bracegirdle et al., 2025; Rahimi-Rizi et al., 2025). However, this is the first study to examine the changes at the low-molecular-weight metabolome (<~650 Da), comprising molecules such as sugars, organic acids, polyols, amino acids, short/medium chain fatty acids, etc., part of primary and secondary metabolism, which has proven to be variable.

Several metabolomic patterns align with ecophysiological expectations for poikilohydric organisms. First, the dominance of arabitol as a fungal storage polyol, followed by ribitol, attributed as the microalgal polyol (**Figure 2A**), fits the role as compatible osmolytes that buffer desiccation-rehydration cycles by equilibrating water potential (Richardson et al., 1968; Honegger, 2012; Pichler et al., 2023), intrinsically linked to environmental drought, the loss of available water due to freezing, temperature increases, high irradiation, etc. The fact that arabitol and ribitol track climate groups and regions (**Figures 5**, **6**) while being constitutively highly abundant (**Figure 2A**) suggests that their steady-state abundance keeps the lichen constitutively prepared for rapid water status changes and at the same time is influenced by the macroclimate (e.g., temperature and precipitation regimes, total solar irradiance, relative humidity) and microclimate site-specific factors (e.g., irradiance exposure, bark composition, recent weather), which modulate their baselines. Notably, their abundances consistently form a distinct cluster apart from other metabolites (**Figure 6**), and the two isomer polyols co-vary by climate and region, aligning with the lichen carbon transfer model. Moreover, both are more abundant under climatic extremes than under milder conditions.

Second, other relevant climate group markers such as glycerol, monoglycerides (1-monopalmitin and glycerol monostearate), and mono/polyunsaturated fatty acids (linoleic and oleic acids) increased significantly toward colder regimes (**Figures 5B**, **6B**). This pattern is consistent with homeoviscous adaptation (Ernst et al., 2016; Los and Leusenko, 2025), in which lipid remodeling preserves membrane fluidity across temperatures, and with the role of glycerol as another compatible solute and a metabolic link connecting lipid and carbohydrate pools. These concepts are novel in lichen fungi, although they have been extensively studied in other biological systems, including microalgae and yeasts (Oren, 2017; Ballweg et al., 2020; Raymond et al., 2020; Paliwal et al., 2021; Hoogerland et al., 2024).

Third, seasonality left a compact yet significant signature. GABA and mannose were the two robust summer discriminants across sites (**Figures 3B, C**). Elevated GABA suggests activation of the GABA shunt, a well-known metabolic valve that, by converting glutamate to this non-proteinogenic amino acid, supports C/N and redox balance under high light and heat stress, a mechanism widely studied in plants and yeast fungi (Cao et al., 2013; Li et al., 2021; Balfagón et al., 2022). By contrast, the increase in mannose is less straightforward to interpret. A plausible mechanistic hypothesis could be that, because growth in *Ramalina* typically peaks in cool-moist periods (autumn-winter) and is comparatively inactive in summer (Boucher and Nash, 1990; Larsson et al., 2014), mannose accumulated upstream of galactomannan biosynthesis, the mannose-rich polymers contributing to *Ramalina* cell walls (Teixeira et al., 1995; Cordeiro et al., 2003; Nascimento Moreira et al., 2015). Such accumulation would be at the same time compatible with the reported roles of hexoses in drought and osmotic stress responses, primarily in vascular plants (Guo et al., 2018; Zhao et al., 2020; Kumar et al., 2021). Notably, these seasonal signals remain consistent across climates (cold, warm, dry, or wet), pointing to novel adaptive mechanisms in lichens.

A final pattern to highlight is that most of the abundant or core metabolites not yet discussed (**Figure 2**) —sucrose, glyceryl-glucoside, sugar alcohols (sorbitol, mannitol, inositol, erythritol), glutamate, and 5-oxoproline— also have recognized roles as osmoprotective compounds, stabilizing proteins, preserving membrane integrity, and mitigating oxidative damage during desiccation and osmotic shock in other organisms (McParland et al., 2021; Mehta and Vyas, 2023; Pepelnjak et al., 2024). Sucrose and glyceryl-glucoside are hallmark compatible solutes upregulated by salt and water stress in cyanobacteria (Klähn and Hagemann, 2011; Kirsch et al., 2019). Polyols are central to desiccation tolerance in lichens and lichen microalgae (Meena et al., 2015; Gasulla et al., 2021; Nazem-Bokaee et al., 2025), and in *R. farinacea*, the accumulation of several polyol types was observed, with arabitol predominating, as discussed above. In addition, glutamate and 5-oxoproline (pyroglutamate), key intermediates of the glutathione cycle, link active osmotic adjustment with oxidative stress defenses (Kranner, 2002; Gasulla et al., 2021). Altogether, the prevalence of these compounds supports a metabolite-based resilience toolkit in *R. farinacea* buffering fluctuations in water potential and oxidative load across seasons and sites, complementing climate-linked lipid remodeling and the seasonal roles of GABA and mannose described above.

At the pathway level, both climate and region analyses (**Figure 7**) converged on the remodeling in alanine/aspartate/glutamate metabolism, indicating co-varying adjustments in amino-acid handling, while the site-specific and climatic variations comprise different processes. Together, the enrichments suggest adjustment in carbon supply (Calvin cycle) and core energy routing (glycolysis/pyruvate/GABA shunt), C-N interconversion (Ala/Asp/Glu; arginine/proline), membrane and transport interfaces (ABC transporters; mannose/galactose), and redox defenses (glutathione), processes expected to tune thallus function to hydration, temperature and irradiance regimes (Canali et al., 2025; Ormond et al., 2025).

This work establishes a continental-scale reference for the *R. farinacea* metabolome, defining a shared metabolic backbone with stable yet factor-specific signatures. Sugar alcohols, most notably arabitol, stand out, alongside other metabolites involved in environmental acclimation, including membrane-lipid remodeling consistent with homeoviscous adaptation. Together, these features are consistent with the lichen’s poikilohydric lifestyle. This research provides a framework for hypothesis-driven studies on the environmental modulation of lichen chemistry, the study of the isolated symbiotic partners metabolome, cross-species and cross-biome comparisons, and opens avenues for (i) integrative multi-omics (transcriptome-metabolome-microbiome) to elucidate the mechanisms underlying the observed metabolome variance; (ii) experimental manipulations (controlled seasonality, moisture, temperature) to test causality; and (iii) bioprospecting of functional metabolites for ecological and biotechnological applications. 

## Data Availability

The original contributions presented in the study are included in the article/**Supplementary Material**. Further inquiries can be directed to the corresponding author.
