# Supplementary material for: Poikilohydria, polyols, and homeoviscosity: lichen metabolomic remodeling across environmental gradients
Source: Front Plant Sci. 2026 May 4;17:1792494. doi: 10.3389/fpls.2026.1792494 (PMC13180806; doi:10.3389/fpls.2026.1792494)
Supplement: Supplementary file 1 [file DataSheet1.pdf]

Supplementary Material

HTML

Supplementary Figure S1. Interactive OpenStreetMap of the *Ramalina farinacea* sampling sites (n = 6 per locality and season). The colored layer reflects the EEA Biogeographical regions classification (2017) and point colors Köppen-Geiger based coding (G1-G5, see Fig. 1 legend). Point description indicates the region, season, Köppen-Geiger code, biogeographical regions (Rivas-Martínez et al., 2017; Moya et al., 2024; European Environment Agency (EEA), 2025), phorophyte, altitude, and distance to sea for each sample.

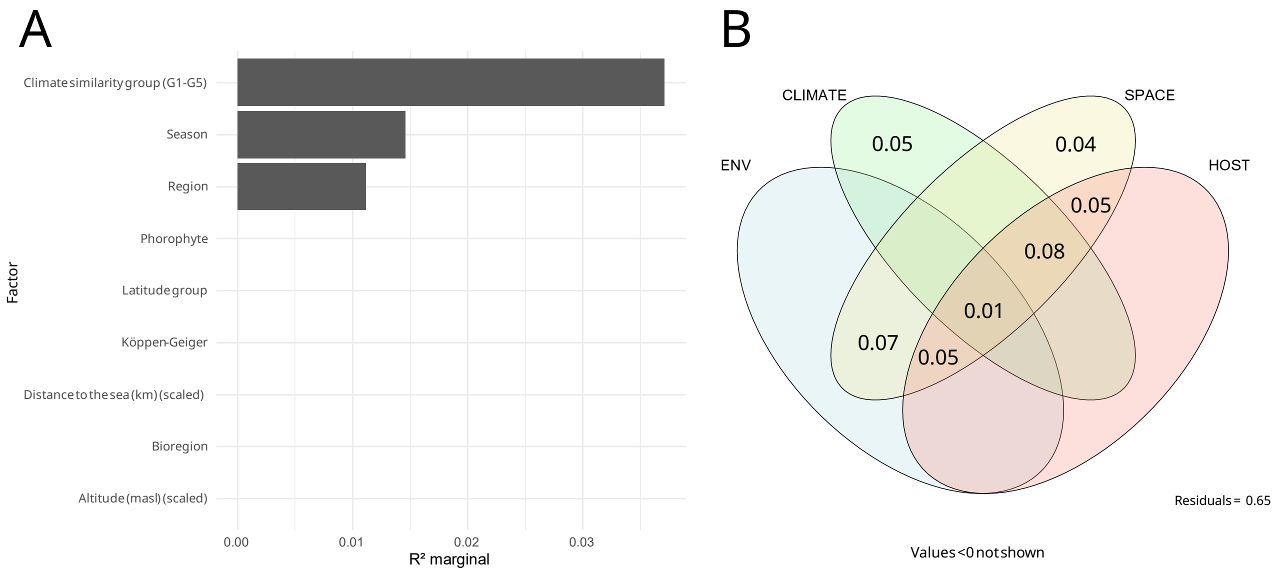

Supplementary Figure S2. Ecological and geographical factors structuring metabolomic variation in *Ramalina farinacea*. (A) PERMANOVA results showing the marginal explanatory power (R^2) of each factor. Factors considered include region, season, Köppen-Geiger climatic zone, climate similarity groups derived from the Köppen-Geiger classification (G1–G5), biogeographical regions (Rivas-Martínez et al., 2017; Moya et al., 2024; European Environment Agency (EEA), 2025), phorophyte, latitude, altitude, and distance to the sea. (B) Variance partitioning analysis (total variance = 1), grouping explanatory variables into four categories: environment (ENV; altitude + sea distance), climate (Köppen–Geiger classification + climate similarity groups), space (spatial structure + region), and host (phorophyte).

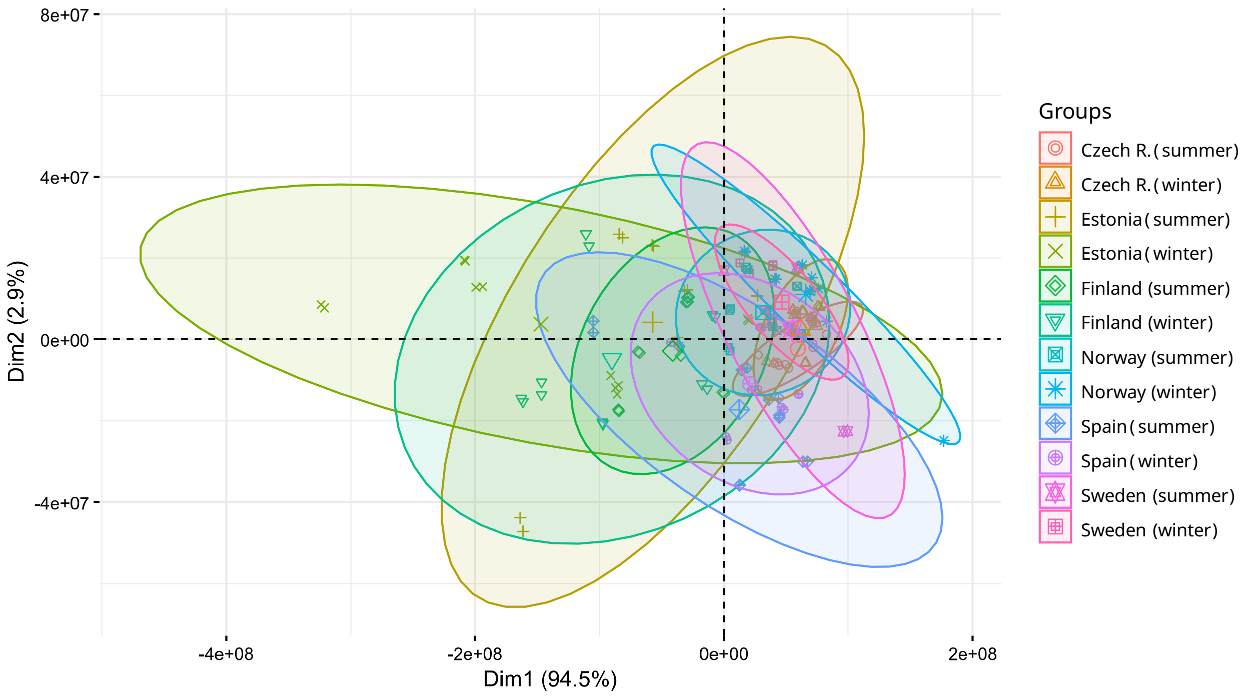

Supplementary Figure S3. Principal Component Analysis (PCA) of metabolomic profiles of *Ramalina farinacea*. Metabolite abundances were obtained by GC-MS from six biological replicates and two technical replicates per region and season. Principal Component 1 (Dim1; 94.5% of total variance) is primarily driven by arabitol abundance (higher on the negative axis), while Principal Component 2 (Dim2; 2.9% of variance) reflects variation mainly associated with ribitol (higher on the negative axis).

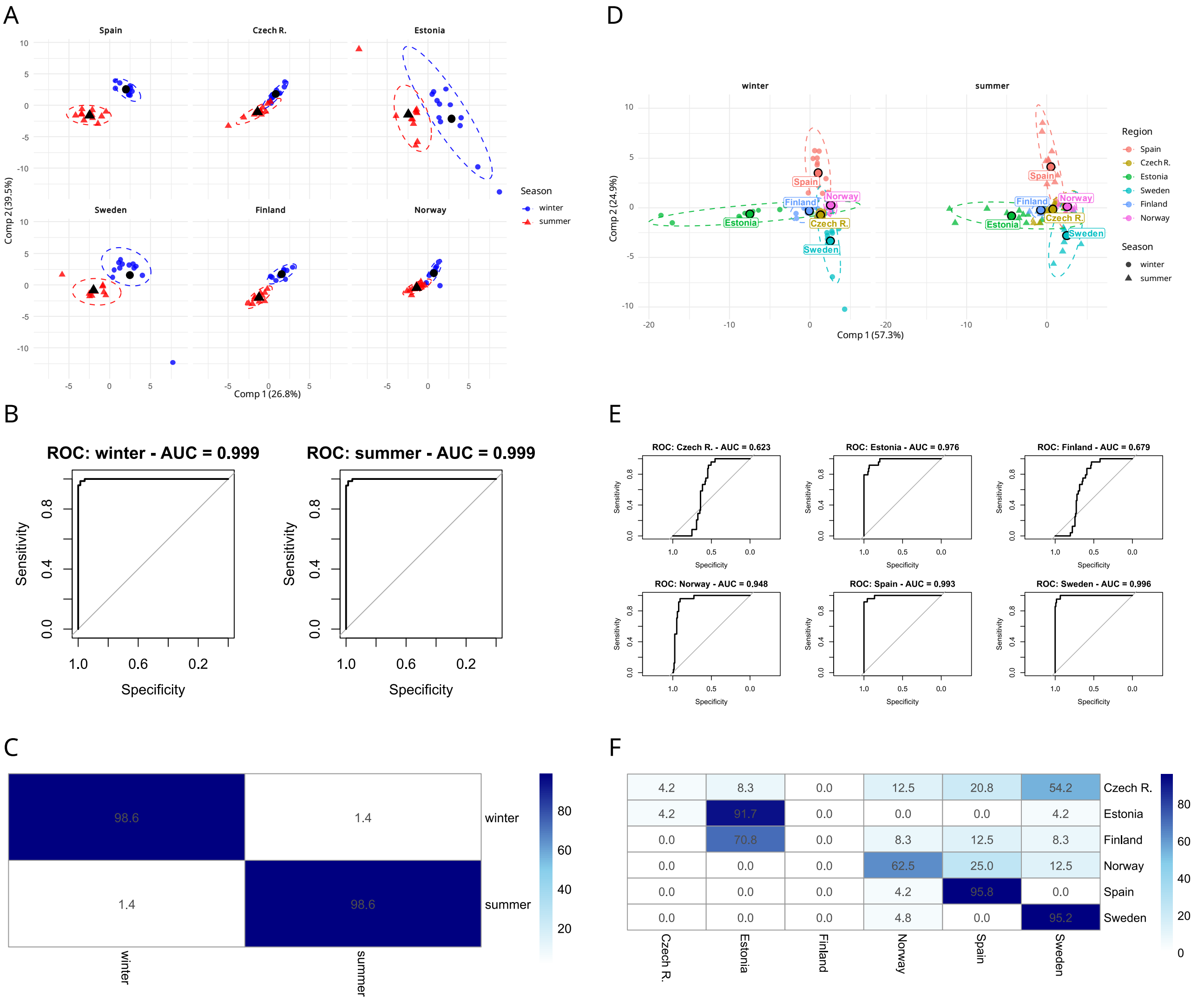

Supplementary Figure S4. Discrimination of *Ramalina farinacea* metabolomes using Partial Least Squares Discriminant Analysis (PLS-DA). (A) PLS-DA score plot of the first two latent components season (faceted by region) and (D) by region (faceted by season: summer and winter). Ellipses indicate 95% confidence intervals for each group, and central points denote group centroids. (B) One-vs-all receiver operating characteristic (ROC) curves with corresponding area under the curve (AUC) values per season and (E) per region. Curves approaching the upper-left corner with AUC > 0.9 indicate excellent discrimination, whereas AUC values around 0.7 reflect moderate separability. (C) Confusion matrix heatmaps from five-fold cross-validation (row-normalized) per season and (F) region models. Diagonal cells show the percentage of correctly classified samples, while off-diagonal cells represent misclassifications.

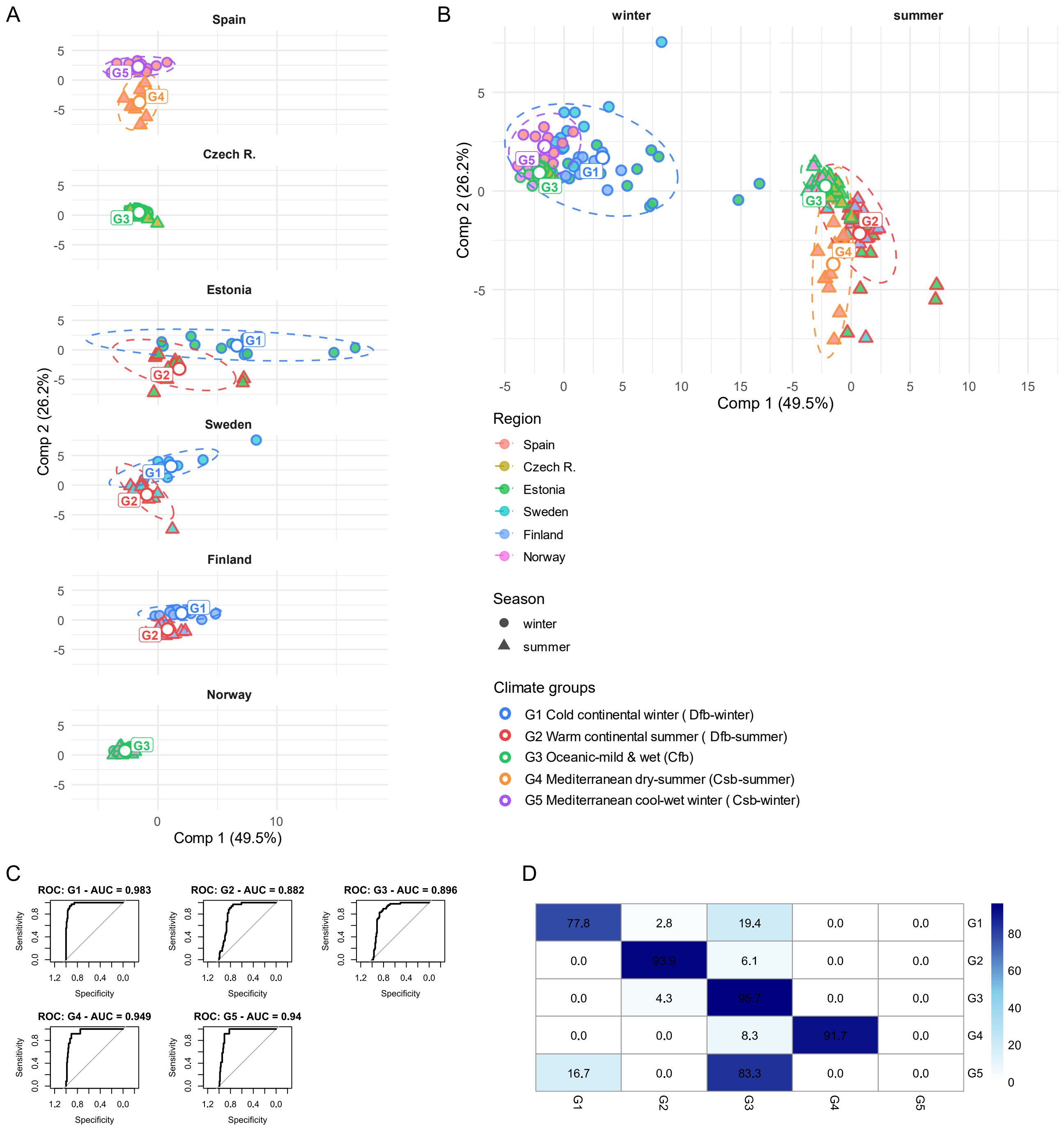

Supplementary Figure S5. Discrimination of *Ramalina farinacea* metabolomes using Partial Least Squares Discriminant Analysis (PLS–DA). (A) PLS–DA score plot of the first two latent components by climate groups, faceted by region, and (B) faceted by season. Ellipses indicate the 95% confidence intervals for each group, and central points denote group centroids. (C) One-vs-all receiver operating characteristic (ROC) curves with corresponding area under the curve (AUC) values per climate group. Curves approaching the upper-left corner with AUC > 0.9 indicate excellent discrimination. (D) Confusion matrix heatmap from five-fold cross-validation (row-normalized) per climate group model. Diagonal cells show the percentage of correctly classified samples, while off-diagonal cells represent misclassifications.

Supplementary Table S1. *Ramalina farinacea* analyzed specimens with collection, classification, and identification details.

Supplementary Table S2. Metabolites annotated in *Ramalina farinacea* with mean normalized areas and standard deviation (SD), grouped by region and season.
